# Promoting respect and preventing mistreatment during childbirth

**DOI:** 10.1111/1471-0528.13750

**Published:** 2015-12-01

**Authors:** JP Vogel, MA Bohren, Ӧ Tunçalp, OT Oladapo, AM Gülmezoglu

**Affiliations:** ^1^ UNDP/UNFPA/UNICEF/WHO/World Bank Special Programme of ResearchDevelopment and Research Training in Human Reproduction (HRP)Department of Reproductive Health and ResearchWorld Health OrganizationGenevaSwitzerland; ^2^ Department of Population, Family and Reproductive HealthJohns Hopkins Bloomberg School of Public HealthBaltimoreMDUSA

One of the key components of efforts to reduce maternal and perinatal morbidity and mortality globally has been to increase rates of facility‐based childbirth. Between 1990 and 2012, the skilled birth attendance rate grew from 57 to 69%, due in part to demand generation through the use of incentives, education and community mobilisation.^1^ Despite these efforts, many women are still unable to reach facilities to give birth because of a range of social, geographical, economic and other barriers. However, many women decide not to seek facility‐based care for childbirth, despite recognising the associated health benefits. This decision is often based on their previous experiences of poor quality care, including poor treatment, abuse, discrimination and neglect while in facilities.^2^ For example, hitting, slapping, physical restraint during childbirth, women and their newborns being detained due to inability to pay, and the use of threats have been documented.^3^ These experiences can, in some instances, constitute a violation of a woman's human rights,^4^ and a violation of the trust women place in caregivers and the health system. It is critical for the maternal health community to ask how it can prevent such mistreatment, and better meet women's socio‐cultural, emotional and psychological needs as part of broader efforts to provide better quality care.

A recent commentary by Tunçalp et al.^5^ described the new World Health Organization (WHO) vision of quality of care for pregnant women and newborns around the time of childbirth, which defined good quality maternal and newborn care as care that is ‘safe, effective, timely, efficient, equitable and people‐centred’. The proposed quality of care framework explicitly considers how care is experienced by women and their families, particularly the importance of ensuring effective communication, respect, dignity and emotional support. These factors are too often overlooked in clinical practice.

Many professional societies, international organisations and civil society groups have recently highlighted the need to address this problem, and promote respectful care practices at birth.^4^, ^6^, ^7^ The White Ribbon Alliance leads a global campaign to promote respectful maternity care.^4^ The International Federation of Gynaecology and Obstetrics, the International Confederation of Midwives, White Ribbon Alliance, the International Paediatrics Association and the WHO have also recently launched the Mother–Baby Friendly Birthing Facilities Initiative, to provide facilities and health systems with actionable steps to improve respectful care at birth.^6^ In September 2014, WHO issued a statement on the prevention and elimination of disrespect and abuse during facility‐based childbirth,^8^ emphasising the rights of every woman to dignified, respectful care during childbirth, and the need for greater action, dialogue, research and advocacy by all health stakeholders on this issue. The statement is now endorsed by over 90 organizations and is available in 15 languages. There are also practical examples from the field on how women's experiences during childbirth can be improved. For example, at the 2015 World Health Assembly Fundacja Rodzić po Ludzku (Childbirth with Dignity Foundation) of Poland were awarded the Saskawa Health Prize for their two decades of work on the ‘Childbirth with Dignity’ campaign.^9^, ^10^ More and better documentation of successful programmes to promote respectful maternity care are needed, so that they can be adapted and adopted in other settings.

One barrier to progress in addressing how women are treated during birth is the lack of globally applicable, agreed upon definitions of what constitutes respectful maternity care and mistreatment during childbirth. For example, the terminology used to describe mistreatment of women at birth in different parts of the world is variable, including terms such as ‘disrespect and abuse’, ‘obstetric violence’ and ‘dehumanised care’.^7^, ^11^, ^12^ This is due in part to cultural and linguistic differences, normative behaviours,^13^ as well as different research methods that have been used to document these experiences.

Defining the mistreatment of women during childbirth is complex. Any definition needs to adequately capture the health, human rights, legal and sociocultural dimensions of this problem. It should consider a range of possible acts (whether intentional or not), the risks (or potential risks) of harm or suffering to women, and that these events can occur in different levels of care. In a recent commentary, Freedman et al.^14^ highlighted the challenges to establishing such a definition, including the need to consider not only women's and provider's experiences, but also intentionality, the role of local societal norms about what constitutes disrespectful or abusive behaviour in different cultures, and how underlying deficiencies in health systems contribute to disrespectful and abusive care. The pathway to a global definition will require further research on what experiences and behaviours constitute mistreatment in different settings from the perspectives of women, providers and other stakeholders. The support of governments, international partners and United Nations agencies will be required to propose an official definition.

To further elucidate this issue, we conducted a mixed methods systematic review of women's experiences of mistreatment during childbirth in facilities, and identified 65 studies from 34 countries, including 11 studies from high‐income countries.^3^ In that review, we reported that mistreatment includes (but is not limited to) experiences of physical, verbal or sexual abuse, stigma and discrimination, failure to meet professional standards of care, ineffective communication, lack of supportive care, detention in facilities, and extortion.^3^ Certain groups of women, such as those of different ethnicities, pregnant adolescents, the poor, migrants and women who are HIV positive, may be more vulnerable to mistreatment than others. Furthermore, the poor physical environment in many facilities, including a lack of privacy and shortages of space, water, electricity, staff, drugs and equipment, can all contribute (directly or indirectly) to negative birth experiences.

Based on our findings in that review, we have proposed the term ‘mistreatment of women during facility‐based childbirth’ as a more broad and inclusive term to describe this phenomenon for three reasons. First, women's own experiences of their maternity care should be central to any description of this phenomenon. Second, some other terms (such as ‘abuse’) that imply a level of intentionality, or ‘acts of commission’ (such as physical or verbal abuse) are not appropriate to describe all forms of mistreatment. The evidence suggests that mistreatment can also be unintentional, or may relate to ‘acts of omission’ (such as long delays due to staff shortages, or a lack of emotionally supportive care from a provider). Third, an inclusive term is needed that captures women's experiences and interactions with staff, the facility environment and the broader health system.

It is important to highlight that preventing mistreatment is not necessarily the same as improving respectful care during birth. Indeed, women may receive care that simultaneously has characteristics that are negative (such as receiving a vaginal examination without privacy) and positive (the provider takes time to clearly communicate the examination findings, and ensures that the woman understands the implications). Although the two concepts are closely linked, interventions to prevent and reduce the mistreatment of women at birth may not necessarily be the same as those that promote respectful maternity care. For example, training providers not to make judgemental or accusatory remarks to women may reduce mistreatment, but may not necessarily make women feel more respected.

Reducing the mistreatment of women in facilities cannot be made without meaningful consideration of the environment in many labour wards worldwide.^15^ Indeed, many providers, striving to provide better quality care, work in settings that can be unsafe, ill‐equipped or overcrowded. They may be unpaid, overworked, and have inadequate support and supervision. It is perhaps unsurprising that such an environment would beget negative experiences, for both pregnant women and for providers themselves.

There are almost no data available to estimate how common the mistreatment of women during childbirth is worldwide. Our review identified three published studies measuring disrespectful and abusive care in maternity facilities, using variable operational definitions and measurement approaches, with estimates ranging from 15 to 98%.^3^ It is clear that evidence‐based, and validated measurement tools that can be used in different countries and settings are needed to quantify the burden in a systematic, comparable way. This would be a crucial step to allow health system stakeholders to identify and address the mistreatment of women when, where and how it occurs.

Furthermore, evidence‐based interventions need to be developed and evaluated, so that health systems can effectively manage this problem. For example, audit and feedback, which have been shown to significantly improve professional practice in health care,^16^ may also be effective in promoting respectful maternity care practices. Once effective interventions (or packages of interventions) are identified, further research will be needed to determine how health facilities and maternity care programmes can efficiently implement and sustain these measures. Much can be learned from related areas, such as the work of the HIV/AIDS community on addressing and reducing stigma and discrimination, and from research on quantifying and preventing other forms of violence, such as gender‐based violence. There is a clear need for a broad and inclusive approach to this issue, one that ensures the active participation of women, communities, healthcare providers, managers, health professional training, education and certification bodies, professional associations, governments and other health systems stakeholders in developing and implementing solutions.

Despite these knowledge gaps, there are several immediate steps that can be taken to promote respectful maternity care practices. For example, WHO recommends that all women should have social support at birth through a companion of choice;^17^, ^18^ there are clear benefits of labour companionship for maternal and newborn outcomes.^19^ However, implementation of this low‐cost, effective intervention remains poor in many countries. Wherever maternity care is delivered, measures to improve women's experiences and support their autonomy and self‐actualisation can be prioritised. This includes clear, respectful, culturally sensitive communication with women and their families regarding their care, as well as efforts to improve standards of privacy, confidentiality and informed consent in facilities. Measures such as labour companion of choice, preferred birth positions, access to food and fluids during labour, provision of information to women on their rights, and equitable, affordable fee structures all warrant implementation. Efforts are also needed to reduce stigma and discrimination of women and their families, and to provide accountability mechanisms for women to seek redress in the event of violations. Providers require training, support and resources so that they can provide good quality, respectful, woman‐centred care. Fundamentally, we must strive to ensure that all women and newborns are treated with the same high standard of respectful, competent care, and are protected from all forms of physical, verbal, emotional and financial abuse while in facilities.

In collaboration with other organisations, WHO aims to play both a research and normative role. In this regard, the UNDP/UNFPA/UNICEF/WHO/World Bank Special Programme of Research, Development and Research Training in Human Reproduction (HRP) has initiated a multi‐country research project to develop and validate evidence‐based tools to measure how women are treated during childbirth.^20^ As part of the vision on quality of care for pregnant women and newborns,^5^ WHO aims to establish standards and indicators of respectful maternal and newborn care, as well as conducting the necessary research to identify, evaluate and implement effective interventions to reduce mistreatment and promote respectful care globally. We call on all members of the maternal health community to contribute to research, implementation and advocacy on this important public health issue.

## Disclosure of interests

Full disclosure of interests available to view online as supporting information.

## Contribution to authorship

This commentary was conceived by JV and MB. JV, MB, OT, OTO and AMG all contributed to the content and development of the article. All authors reviewed and agreed to the final version of this manuscript. This article represents the views of the named authors only.

## Details of ethics approval

No ethics approval required.

## Funding

The WHO technical consultation ‘Preventing Disrespect and Abuse in Facilities During Childbirth’ (November 2013) and the research work of WHO's Department of Reproductive Health and Research on this issue is supported by the United States Agency for International Development (USAID) (USAID Amendment #20, WHO Consolidated Grant GHA‐G‐00‐09‐0003).

## References

[1] United Nations . Millennium Development Goals Report 2014. New York: United Nations, 2014.

[2] Bohren MA , Hunter EC , Munthe‐Kaas HM , Souza JP , Vogel JP , Gulmezoglu AM . Facilitators and barriers to facility‐based delivery in low‐ and middle‐income countries: a qualitative evidence synthesis. Reprod Health 2014;11:71. 25238684 10.1186/1742-4755-11-71 PMC4247708

[3] Bohren MA , Vogel JP , Hunter EC , Lutsiv O , Makh SK , Souza JP , et al. The Mistreatment of Women during Childbirth in Health Facilities Globally: a Mixed‐Methods Systematic Review. PLoS Med 2015;12:e1001847. doi:10.1371/journal.pmed.1001847. 26126110 10.1371/journal.pmed.1001847 PMC4488322

[4] White Ribbon Alliance . Respectful Maternity Care: The Universal Rights of Childbearing Women. Washington DC: White Ribbon Alliance, 2011.

[5] Tunçalp Ӧ , Were WM , MacLennan C , Oladapo OT , Gulmezoglu AM , Bahl R , et al. Quality of care for pregnant women and newborns—the WHO vision. BJOG 2015;122:1045–9. 25929823 10.1111/1471-0528.13451 PMC5029576

[6] Lalonde AB , Miller S . Mother‐baby friendly birthing facilities initiative. Int J Gynaecol Obstet 2015;128:93–4. 25497051 10.1016/j.ijgo.2014.11.002

[7] D'Gregorio RP . Obstetric violence: a new legal term introduced in Venezuela. Int J Gynaecol Obstet 2010;111:201–2. 20926074 10.1016/j.ijgo.2010.09.002

[8] World Health Organization . WHO Statement on the Prevention and Elimination of Disrespect and Abuse during Facility‐based Childbirth [Internet]. Geneva: World Health Organization, 2014 [http://apps.who.int/iris/bitstream/10665/134588/1/WHO_RHR_14.23_eng.pdf?ua=1&ua=1].

[9] World Health Organization . Raising the voices of pregnant women in Poland [Internet]. [www.who.int. Geneva. [www.who.int/features/2015/childbirth-dignity-poland/en/]. Accessed 20 August 2015.

[10] Fundacja Rodzic po Ludzku, editor. Fundacja Rodzic po Ludzku (Childbirth with Dignity Foundation) [Internet]. [www.gdzierodzic.info/]. Accessed September 2015.

[11] Bowser D , Hill K . Exploring Evidence for Disrespect and Abuse in Facility‐based Childbirth: report of a landscape analysis. USAID/TRAction Project. Washington DC: USAID, 2010.

[12] Misago C , Kendall C , Freitas P , Haneda K , Silveira D , Onuki D , et al. From ‘culture of dehumanization of childbirth’ to ‘childbirth as a transformative experience’: changes in five municipalities in north‐east Brazil. Int J Gynaecol Obstet 2001;75:S67–72. 11742645

[13] Freedman LP , Kruk ME . Disrespect and abuse of women in childbirth: challenging the global quality and accountability agendas. Lancet 2014;384:e42–4. 24965825 10.1016/S0140-6736(14)60859-X

[14] Freedman LP , Ramsey K , Abuya T , Bellows B , Ndwiga C , Warren CE , et al. Defining disrespect and abuse of women in childbirth: a research, policy and rights agenda. Bull World Health Organ 2014;92:915–7. 25552776 10.2471/BLT.14.137869 PMC4264393

[15] Bradley S , Kamwendo F , Chipeta E , Chimwaza W , de Pinho H , McAuliffe E . Too few staff, too many patients: a qualitative study of the impact on obstetric care providers and on quality of care in Malawi. BMC Pregnancy Childbirth 2014;15:65. 10.1186/s12884-015-0492-5 PMC4377843 25880644

[16] Ivers N , Jamtvedt G , Flottorp S , Young JM , Odgaard‐Jensen J , French SD , et al. Audit and feedback: effects on professional practice and healthcare outcomes. Cochrane Database Syst Rev 2011;6:CD000259. 10.1002/14651858.CD000259.pub3 PMC11338587 22696318

[17] World Health Organization . WHO Recommendations for Augmentation of Labour. Geneva: World Health Organization; 2014. 25506951

[18] World Health Organization . WHO Recommendations on Health Promotion Interventions for Maternal and Newborn Health [Internet]. Geneva: World Health Organization, 2015 [www.who.int/maternal_child_adolescent/documents/health-promotion-interventions/en/] 26180864

[19] Hodnett ED , Gates S , Hofmeyr GJ , Sakala C . Continuous support for women during childbirth. Cochrane Database Syst Rev 2013;7:CD003766. 23857334 10.1002/14651858.CD003766.pub5

[20] Vogel JP , Bohren MA , Tunçalp O , Oladapo OT , Adanu RM , Baldé MD , et al. How women are treated during facility‐based childbirth: development and validation of measurement tools in four countries ‐ phase 1 formative research study protocol. Reprod Health 2014;12:60. 10.1186/s12978-015-0047-2 PMC4510886 26198988

